# Epidemiological Surveillance Reveals the Rise and Establishment of the Omicron SARS-CoV-2 Variant in Brazil

**DOI:** 10.3390/v15041017

**Published:** 2023-04-20

**Authors:** Joice do Prado Silva, Aline Brito de Lima, Luige Biciati Alvim, Frederico Scott Varella Malta, Cristiane Pinheiro Toscano Brito Mendonça, André Henrique Barbosa de Carvalho, Jéssica Silqueira Hickson Rios, Paula Luize Camargos Fonseca, Daniel Costa Queiroz, Luíza Campos Guerra de Araújo e Santos, Alessandro Clayton de Souza Ferreira, Renan Pedra de Souza, Renato Santana de Aguiar, Danielle Alves Gomes Zauli

**Affiliations:** 1 Departamento de Pesquisa & Desenvolvimento, Instituto Hermes Pardini, Belo Horizonte 31270-901, Brazil; 2 Departamento de Produtos e Inovação, Instituto Hermes Pardini, Belo Horizonte 31270-901, Brazil; 3 Laboratório de Biologia Integrativa, Departamento de Genética, Ecologia e Evolução, Instituto de Ciências Biológicas, Universidade Federal de Minas Gerais, Belo Horizonte 31270-901, Brazil; 4 Instituto D’Or de Pesquisa e Ensino (IDOR), Rio de Janeiro 22281-100, Brazil

**Keywords:** SARS-CoV-2, variants, epidemiologic surveillance, COVID-19, viral load, symptoms

## Abstract

The introduction of SARS-CoV-2 variants of concern (VOCs) in Brazil has been associated with major impacts on the epidemiological and public health scenario. In this study, 291,571 samples were investigated for SARS-CoV-2 variants from August 2021 to March 2022 (the highest peak of positive cases) in four geographical regions of Brazil. To identify the frequency, introduction, and dispersion of SARS-CoV-2 variants in 12 Brazilian capitals, VOCs defining spike mutations were identified in 35,735 samples through genotyping and viral genome sequencing. Omicron VOC was detected in late November 2021 and replaced the Delta VOC in approximately 3.5 weeks. We estimated viral load differences between SARS-CoV-2 Delta and Omicron through the evaluation of the RT-qPCR cycle threshold (Ct) score in 77,262 samples. The analysis demonstrated that the Omicron VOC has a lower viral load in infected patients than the Delta VOC. Analyses of clinical outcomes in 17,586 patients across the country indicated that individuals infected with Omicron were less likely to need ventilatory support. The results of our study reinforce the importance of surveillance programs at the national level and showed the introduction and faster dispersion of Omicron over Delta VOC in Brazil without increasing the numbers of severe cases of COVID-19.

## 1. Introduction

Coronavirus disease 2019 (COVID-19) is caused by severe acute respiratory syndrome coronavirus 2 (SARS-CoV-2). In the last three years, SARS-CoV-2 has infected almost 757 million people and been responsible for over 6 million deaths worldwide [1]. Brazil reported more than 36 million cases and about 698 thousand deaths [2]. Nonetheless, the vaccination programs demonstrated high efficiency to combat SARS-CoV-2 infection and dissemination [3,4]. Despite vaccination programs, individual protection measures helped contain the SARS-CoV-2 spread worldwide [5,6,7].

To monitor new SARS-CoV-2 variants according to their potential impact on virus spread, disease severity, vaccine performance, diagnostic tools, and others, the World Health Organization (WHO) created the classification of Variants of Interest (VOIs), Variants of Concern (VOCs), Variants under Monitoring (VUMs), and Formerly Monitored Variants (FMVs) [8]. While VOIs, VUMs, and FMVs have unclear impacts on human health [9], the VOCs are associated with immune escape and have the potential for higher transmissibility and virulence [10,11]. Thus far, the WHO has designated five VOCs: Alpha (B.1.1.7) [12]; Beta (B.1.351) [13]; Gamma (P.1) [14,15]; Delta (B.1.617.2) [16]; and Omicron (B.1.1.529) [17], identified in the United Kingdom, South Africa, Brazil, India, and South Africa, respectively. The VOC’s establishment has also been responsible for upsurges in cases and deaths in Brazil [18], for example, the collapse of the health system observed in Manaus in early 2021 by Gamma VOC [14,19], the increase in the number of severe cases due to Delta VOC in mid-2021 [20], and finally, the rise in the number of cases due to Omicron VOC that promoted the two last waves of COVID-19 in 2022 [21].

The Omicron VOC, first reported in November 2021 [22,23], contains at least 32 mutations in the spike protein, 10 of which are in the receptor-binding domain, which represents twice as many as the Delta variant [24]. One of the main mutations is the 69–70 deletion, which prevents the oligonucleotide probe in some RT-qPCR kits from binding to its target sequence, leading to what has been called S gene target failure (SGTF). The SGTF is a marker for Omicron and Alpha detection, but it is absent in the Delta VOC [25]. Nowadays, several Omicron sublineages have been detected, including BA.1, BA.1.1, BA.2, BA.3, BA.2.12.1, BA.4, and BA.5. Indeed, several Omicron recombinants have been reported more recently. According to previous studies, Omicron may be less severe than other VOCs. However, it displays higher humoral immunity evasion and rapid dissemination abilities [26,27]. Although cases attributed to Omicron VOC are generally classified as milder by health authorities, it is important to maintain continuous epidemiological surveillance since more aggressive variants may emerge and need to be identified as quickly as possible.

Thus, several scientific initiatives around the world have been contributing to the generation of information about the COVID-19 pandemic situation. These data include monitoring of prevalent variants, incidence, number of deaths, descriptions of mutations, the clinical relevance of variants, vaccination impact, and other aspects [28,29,30,31]. In this context, our group created a SARS-CoV-2 variants monitoring initiative in Brazil that recently described the replacement of Gamma by Delta SARS-CoV-2 VOCs between April and October 2021 [28]. Hence, the present study aimed to evaluate the transition of Delta to Omicron SARS-CoV-2 VOCs in Brazilian territory from August 2021 to March 2022, symptomatology features, and viral load in a new scenario of increasing rates of transmission of COVID-19.

## 2. Materials and Methods

### 2.1. Study Design

This study was approved by the Research Ethics Committee (CAAE-33202820.7.1001.5348) with the exemption of the individual participants’ consent form to investigate virus whole genome sequences. Data analysis included a descriptive sample profile, genomic surveillance of SARS-CoV-2 by reverse transcription-quantitative polymerase chain reaction (RT-qPCR) genotyping analysis and whole-genome sequencing, determination of Omicron VOC viral load by measuring RT-qPCR cycle threshold (Ct) score, and a meta-analysis of the symptomatology dynamics of Omicron VOC.

### 2.2. Genomic Surveillance by RT-qPCR Genotyping and Whole-Genome Sequencing

#### 2.2.1. Descriptive Sample Profile and SARS-CoV-2 Variants Genotyping by RT-qPCR

A total of 210,941 nasopharyngeal swab samples were evaluated by the SARS-CoV-2 diagnostic test by RT-qPCR performed in the Pardini Group laboratory. Total viral RNA isolation was performed as previously described, and RT-qPCR was performed by the TaqPath COVID-19 CE-IVD RT-PCR kit (Thermo Fisher, Waltham, MA, USA) according to the manufacturer’s instructions for evaluation of three distinct viral targets: the N, ORF1ab, and S genes [28,32]. The samples were collected from 12 Brazilian capitals: Belém, Belo Horizonte, Boa Vista, Brasília, Fortaleza, Goiânia, Macapá, Manaus, Palmas, Porto Velho, Rio de Janeiro, and São Paulo, from 29 August 2021 to 5 March 2022, corresponding to 35/2021 to 09/2022 epidemiological weeks. The data were then categorized into positive and negative results. The positive samples were grouped by sex (male and female) and age cluster (0 to 9 years; 10 to 19 years; 20 to 29 years; 30 to 39 years; 40 to 49 years; 50 to 59 years; and ≥60 years). The SARS-CoV-2 variant genotyping in this study was performed according to the SGTF detection method [25]. SGTF were considered only in samples with CT values < 26 for any other viral gene (N or ORF1ab) to exclude samples with SGTF profiles due to lower viral input in primary samples. Therefore, SGTF samples were named Omicron and non-SGTF samples were named Delta, according to the expected frequencies of variants in the evaluated time. The results were plotted on moving average graphs using the ggplot2 [33] package of the R program (R version 4.1.1; R Foundation for Statistical Computing, Vienna, Austria). The datasets and codes used are available in Supplementary File S1.

#### 2.2.2. SARS-CoV-2 Genome Sequencing

Viral genome sequencing was performed on 109 SARS-CoV-2 positive samples with a Ct value < 26. Nucleic acids were extracted using the MagMAX Viral/Pathogen Nucleic Acid Isolation Kit (Thermo Fisher, Waltham, MA, USA). Sequencing libraries were prepared by the QIAseq FX DNA Library Prep kit (QIAGEN, Hilden, NRW, Germany) and ARTIC V4.0 multiplex primer set to Delta or ARTIC V4.1 multiplex primer set to Omicron. Genomes were sequenced on the MiSeq platform (Illumina, San Diego, CA, USA) with v3 cartridges (600 cycles) following the manufacturer’s instructions. For each of the sample processing steps (cDNA synthesis, viral genome amplification, and library preparation), a negative control was included.

#### 2.2.3. Viral Genome Assembly

Raw sequence data files were processed using the pipeline ViralUnit, as described previously [34]. Briefly, reads were filtered and trimmed using Trimmomatic (minimum read quality score of 30) [35]. The remaining reads were mapped to the reference SARS-CoV-2 genome (NCBI GenBank accession NC_045512) using Minimap2 [36], with unmapped reads discarded. Mapping files were then indexed and sorted with SAMtools v1.12 [37]. To infer the consensus genome sequences, BCFtools v1.12 [38] was used. Finally, BEDtools v2.30.0 [39] was used to mask low-depth sites (<10× coverage). To ensure the accuracy of variant calls, only genomes with >70% genome coverage were included. All consensus genome sequences characterized in this study have been deposited on GISAID and are publicly available (Supplementary Table S1).

#### 2.2.4. Lineage Classification and Phylogenetic Analysis

The consensus sequences were classified using the Pangolin tool v.4.1.3 [40] and the NextClade web application v.2.3.0 [41]. A dataset (*n* = 1640) containing public reference genomes classified by Nextstrain [42] as Omicron lineages (*n* = 1531) (acknowledgments are available in Supplementary File S5) and genomes generated in this study (*n* = 109) was aligned using Minimap2 [36]. A Maximum Likelihood phylogeny was inferred using the IQ-tree program v2.0.3 [43] under the GTR+F+I+G4 model to corroborate the classification generated by Pangolin and NextClade [44]. The support value of the branches was evaluated using the Bootstrap test with 1000 pseudoreplicates. The dataset and script are available in Supplementary File S2.

### 2.3. Ct Value and Viral Load

The RT-qPCR test Ct values were used to assess the viral load differences in the upper respiratory tract between SARS-CoV-2 Delta and Omicron variants. In this approach, the Ct score is inversely proportional to the amount of viral load available in the biological samples, suggesting the transmission rates associated with the variants evaluated [15,45,46].

Data from 77,262 SARS-CoV-2 positive patients collected between 29 August 2021, and 5 March 2022, in 12 Brazilian capitals were evaluated. The Ct values for N and ORF1ab viral targets were obtained as described previously [28]. To estimate differences in the distribution of Ct values in periods dominated by different VOCs, the data were anonymized and categorized into Delta or Omicron groups. Each group included only the Ct value of the epidemiological weeks when a given lineage exhibited frequency above 90% at the time of collection. The dominance period was defined for each capital and period intermediate frequency; those that did not reach the dominance cut-off were not analyzed (Supplementary Table S2).

The variant effect on the Ct score was estimated at the national level and for each capital using a linear regression model, and the median Ct difference between groups was assessed using the Student’s *t*-test. The statistical analyses were conducted as implemented in the R software, and results were plotted using the ggplot2 packages (R version 4.2.2; R Foundation for Statistical Computing, Vienna, Austria). The dataset and code used are available in Supplementary File S3 [33].

### 2.4. Symptomatology Meta-Analysis

The Severe Acute Respiratory Syndrome (SARS) database made available through the Brazilian Health Surveillance Secretariat was used to obtain clinical data of hospitalized patients with suspected COVID-19 in the Brazilian Unified Health System (SUS) [47]. A total of 17,586 medical records of patients with positive SARS-CoV-2 RT-qPCR and complete registration information from 10 Brazilian capitals (Belém, Belo Horizonte, Boa Vista, Fortaleza, Goiânia, Macapá, Manaus, Porto Velho, Rio de Janeiro, and São Paulo) were selected in the evaluated period. The groups were clustered according to the periods in which Omicron (case) and Delta (control) VOCs had a minimum prevalence of 90% in each capital. The meta-analysis (random and common effects) and the heterogeneity among capitals were calculated for the following clinical data: need for ventilatory support, intensive care unit (ICU) admission, and death [28]. The statistical analysis was performed using the R software (R version 4.1.3; R Foundation for Statistical Computing, Vienna, Austria). The dataset and code are available in Supplementary File S4.

## 3. Results

### 3.1. The Rapid Replacement and Establishment of Omicron VOC in Brazilian Territory

**Figure 1:**
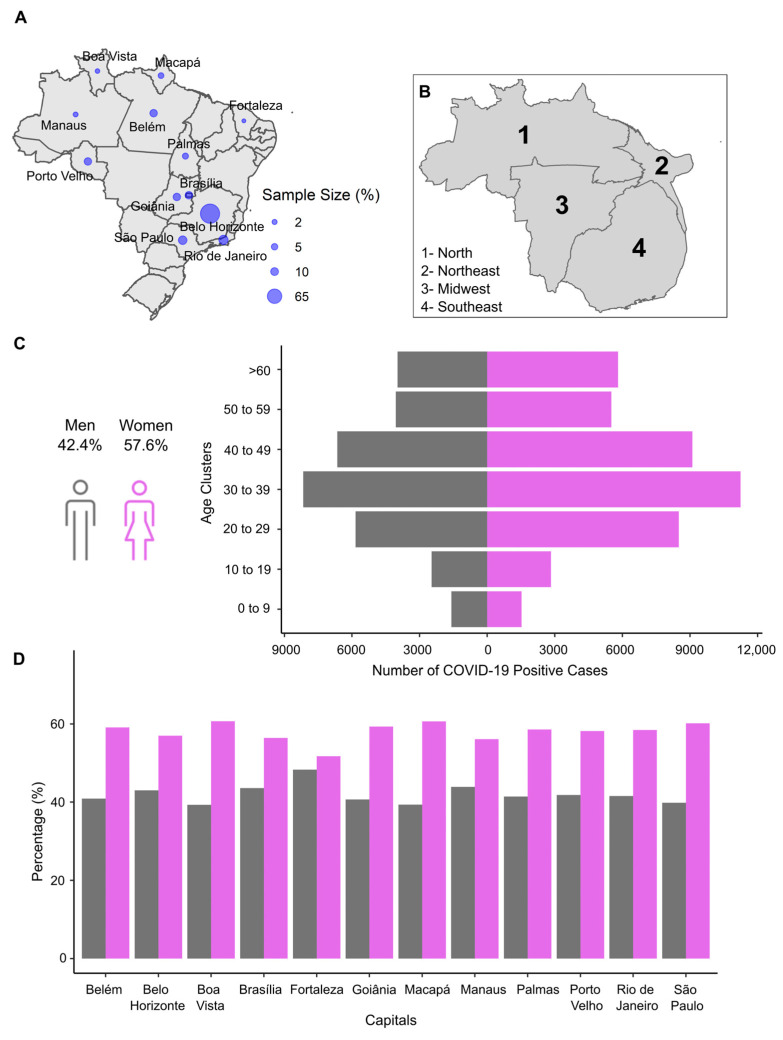
Descriptive analysis of the study design. (**A**) Twelve capitals distributed along four macro-regions of Brazil were included in this study. A total of 77,262 samples with confirmed positive COVID-19 diagnoses were evaluated. The blue circle size represents the proportion of positive samples in each Brazilian capital; (**B**) the cartogram of sample representativeness according to the population of each Brazilian region; (**C**) the sex and age profile of the study sample (data were grouped by sex and age clusters); and (**D**) the sex profile by Brazilian capital of the study sample. The gray and magenta bars correspond to men’s and women’s records, respectively.

A total of 291,571 nasopharyngeal swab samples were collected from patients with symptoms characteristic of COVID-19 or who had contact with COVID-19 positive patients. All samples were tested by RT-qPCR, and of the total, 77,262 had a confirmed positive diagnosis. With one of the largest samples taken in Brazil, this study covered 12 capitals. Belo Horizonte was the capital with the highest number of samples (*n* = 49,149; positive rate = 23.97%), followed by Rio de Janeiro (*n* = 6590; positive rate = 31.16%), São Paulo (*n* = 4909; positive rate = 36.76%), Goiânia (*n* = 3248; positive rate = 25.32%), Brasília (*n* = 3149; positive rate = 27.68%), Belém (*n* = 3112; positive rate = 39.17%), Porto Velho (*n* = 3059; positive rate = 37.59%), Palmas (*n* = 1509; positive rate = 29.73%), Macapá (*n* = 1131; positive rate = 44.79%), Manaus (*n* = 590; positive rate = 32.49%), Boa Vista (*n* = 468; positive rate = 36.42%), and Fortaleza (*n* = 348; positive rate = 34.18%) (Figure 1A). The Midwest and Southeast regions had the highest sampling rates (number of samples/population size in each region) compared to the other studied areas (Figure 1B). The number of women with positive results (57.6%) was higher than men’s (42.4%) in all capitals and age clusters, with an exception for the 0–9-year-old group (Figure 1C). People ranging in age from 20 to 49 years old represented the group with the highest index of positivity (64.1%; 49,501/77,262). There were no large fluctuations in the proportion of male and female individuals by Brazilian capital (Figure 1D).

We used the SGTF profile to identify possible Omicron samples. For that, we only considered positive RT-qPCR samples with CT values < 26 for any viral gene (N and ORF1ab) using the TaqPath COVID-19 CE-IVD RT-PCR kit. With this strategy, we guarantee that the SGTF profile was due to mutations in the 69/70 position of the S gene, ruling out the influence of lower viral input on primary samples in our analyses. This analysis comprised a final dataset of 35,735 positive samples.

**Figure 2:**
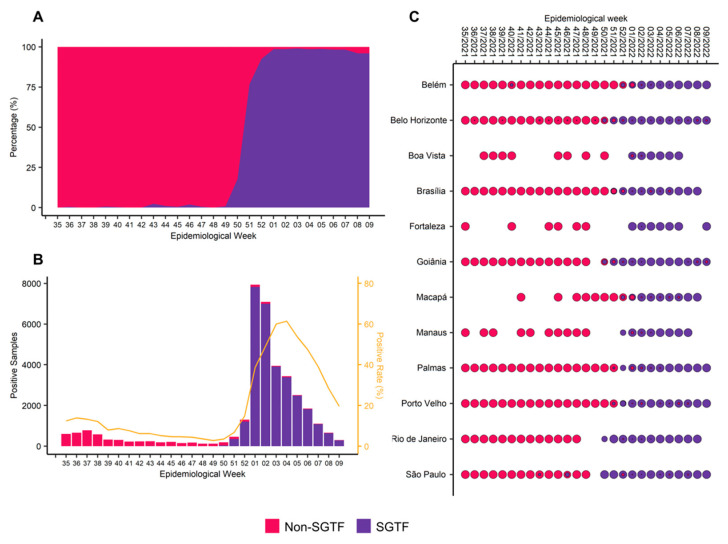
Monitoring of Delta and Omicron VOCs in Brazil. Delta and Omicron variants were represented by red and dark purple colors, respectively. (**A**) Percentage of non-SGTF and SGTF samples per epidemiological week in 35,735 samples across Brazil. (**B**) Absolute numbers of non-SGTF and SGTF profiles in the positive RT-PCR samples per epidemiological week and positive rate; (**C**) Transition period between non-SGTF and SGTF samples by capital and epidemiological week. Blank spaces indicate the absence of samples in a particular capital within the specified epidemiological week.

In the first week of study monitoring (epidemiological week 35), it was observed that Delta VOC (non-SGTF) was already predominant (>90% frequency) in all evaluated capitals, which remained until mid-December 21 (epidemiological week 50) (Figure 2A). Although we detected a remnant of SGTF-positive samples since epidemiological week 36 in Belo Horizonte, the exponential increase of SGTF-positive cases (Omicron VOC) only occurred after epidemiological week 49. This increase in the number of cases of infected individuals resulted in the rapid establishment and predominance of Omicron VOCs to the detriment of Delta VOCs (Figure 2B). The rise of Omicron cases occurred first in the Southeast region (São Paulo, Rio de Janeiro, and Belo Horizonte) and Goiânia (the Midwest region), where Omicron became predominant in mid-December 2021. On the other hand, in Fortaleza (the northeast region), Brasília (the Midwest region), Belém, Boa Vista, Macapá, Manaus, Palmas, and Porto Velho (all in the north region), the rise of Omicron cases occurred slowly, reaching the predominance in January 2022, between epidemiological weeks 1–2 (Figure 2C).

### 3.2. Sequencing Metrics, Classification, and Phylogeny

**Figure 3:**
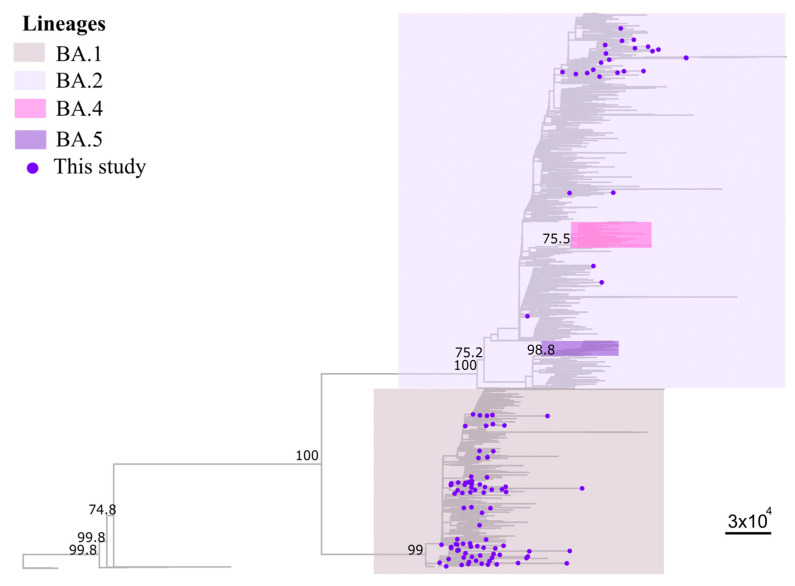
Phylogenetic reconstruction of SARS-CoV-2 during the fourth wave of SARS-CoV-2 in Brazil (epidemiological weeks 35/2021 to 09/2022). The dataset was constructed based on the genomes used by Nextclade as references for Omicron classification (*n* = 1531). Maximum likelihood phylogenetic tree inferred from our dataset to confirm lineage classification. Purple-tipped shapes indicate genomes generated in our study (*n* = 109). Node values correspond to bootstrap values. The clades corresponding to the variants BA.1, BA.2, BA.4, and BA.5 are highlighted in gray, lilac, pink, and purple, respectively.

During the study monitoring, samples were randomly selected and directed to whole-genome sequencing to confirm the variant detected by the SGTF method (0.31%; 109/35,735). The median genome coverage was 87.6% (63.2 to 98.5%), and the median sequencing depth was 2749.97× (1028.22 to 5239.48×). The sequences were initially analyzed by Pangolin and NextClade tools. Of these, 84 (77.1%) genomes were classified as belonging to the BA.1 clade: BA.1 (53); BA.1.1 (11); BA.1.1.16 (1); BA.1.1.18 (1); BA.1.14 (1); BA.1.14.1 (7); BA.1.14.2 (1); BA.1.15 (1); BA.1.16 (3); BA.1.22 (4); BA.1.7 (1); while 25 (22.9%) genomes were classified as belonging to the BA.2 clade: BA.2 (24) and BA.2.3 (1). Sequencing metrics and Pangolin/Nextclade classification are available in Supplementary Table S3. To corroborate the results found in the Pangolin/Nextclade classification, we performed a maximum likelihood analysis. Our phylogenic reconstruction confirmed the Pango/NextClade for all samples analyzed (Figure 3).

### 3.3. Omicron-Infected Individuals Present a Lower Viral Load in the Upper Respiratory Tract

To determine whether the spread of the Omicron VOC was related to the induction of a high viral load in the upper respiratory tract compared to the Delta VOC, we distributed positive RT-qPCR Ct values obtained from infected patients in two groups, considering the dominant variant in the studied period. The final dataset consisted of 72,885 samples distributed in the Omicron group (*n* = 63,420) or Delta group (*n* = 9465).

**Figure 4:**
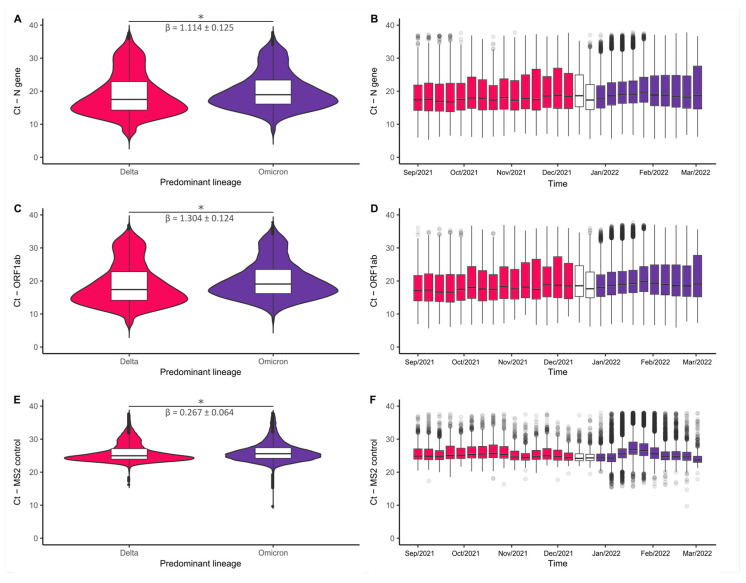
Comparative analysis of RT-qPCR Ct values between Delta and Omicron SARS-CoV-2 VOC dominance periods. Colors represent the dominant variant, with a frequency above 90% in the analyzed period, being Delta (red) or Omicron (dark purple). Violin plots displaying the distribution of Ct data according to the predominant lineage for the viral target genes N (**A**) and ORF1ab (**C**), and the MS2 internal process control (**E**). Boxplots display Ct value variation along the epidemiological weeks comprised in the study period (August 29, 2021–March 5, 2022) for the viral target genes N (**B**), ORF1ab (**D**), and MS2 internal control (**F**). A statistical comparison between periods denotes that Omicron VOC might be associated with lower viral loads in the upper respiratory tract than Delta VOC infection. White bars represent the first quartile, the median and the third quartile. Black circles represent the outliers * *p* < 0.001.

Comparative analysis by linear regression indicated that the Omicron VOC induces an increase in Ct values in the studied viral targets (N: β = 1.114, 95% CI = ± 0.125, *p* < 0.001; ORF1ab: β = 1.304, 95% CI = ± 0.124; *p* < 0.001). While the median Ct in the Omicron group was 18.94 (N) and 19.07 (ORF1ab), the Delta group had lower values, 17.51 (N) and 17.40 (ORF1ab) (Figure 4A,C). A residual effect was also inferred for the MS2 exogenous control target (median Ct: Omicron = 25.58, Delta = 24.97; β = 0.267, 95% CI = ± 0.064, *p* < 0.001); however, its effect is almost 5-fold smaller than that calculated for the viral targets (N gene and ORF1ab) (Figure 4E). The Omicron VOC effect on viral load can be followed in Ct value time series along epidemiological weeks (Figure 4B,D,F). This trend was observed in most of the Brazilian capitals, suggesting lower viral loads of Omicron VOC assigned positive in the upper respiratory tract compared with Delta VOC (Supplementary Table S4).

### 3.4. Effect of SARS-CoV-2 Variants on Symptomatology and Clinical Data

**Figure 5:**
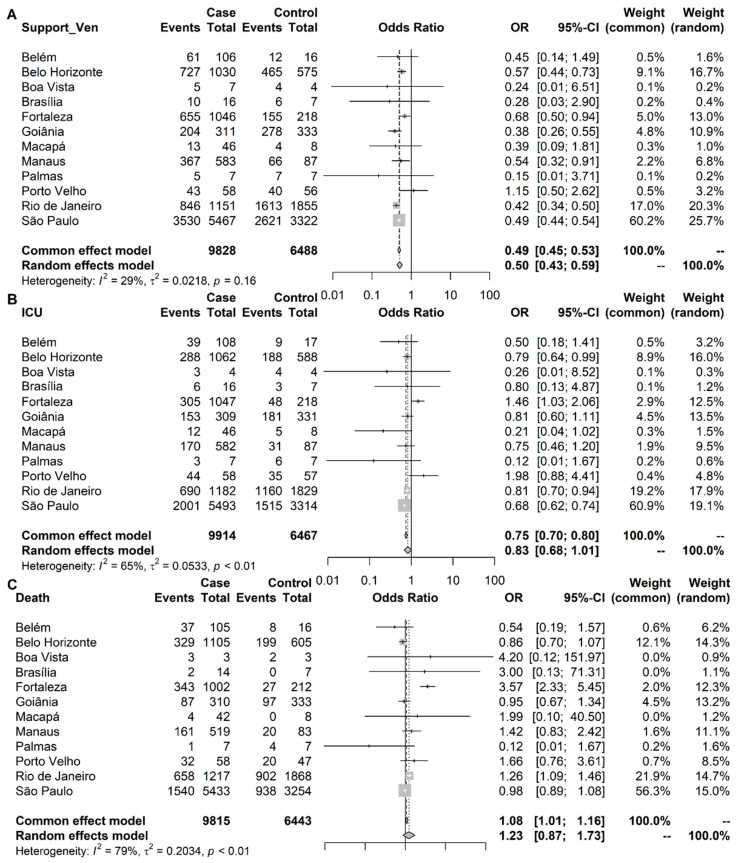
Forest plot of effect sizes measured between Omicron (case) and Delta (control) groups in COVID-19 patients for: (**A**) need for ventilatory support: 9828 cases and 6488 controls; (**B**) intensive care unit admission: 9914 cases and 6467 controls; and (**C**) death: 9815 cases and 6443 controls, in a meta-analysis of 12 different Brazilian capitals according to the periods in which Omicron (case) and Delta (control) VOCs had a minimum prevalence of 90% in each capital city.

To explore symptomatologic features in hospitalized patients with suspected COVID-19, we evaluated data from the Brazilian SRAG database in periods dominated by Delta or Omicron VOCs. Three sets of clinical data were accessed: the need for ventilatory support (16,316 patients), ICU admission (16,381 patients), and death (16,258 patients). The meta-analysis results showed that the need for ventilatory support was less frequent in the Omicron group (Odds Ratio—OR: 0.50; 95% CI: 0.43–0.59) than in the Delta group (Figure 5A). There was no statistically significant difference between the Omicron and Delta groups regarding the frequency of ICU admissions and deaths (Odds Ratio—OR: 0.83; 95% CI: 0.68–1.01 and OR: 1.23; 95% CI: 0.87–1.73, respectively) (Figure 5B,C).

## 4. Discussion

From the onset of the pandemic, epidemiologic surveillance programs have allowed the monitoring of viral spread and VOC emergence, making them one of the forefronts of COVID-19 combat around the world [48]. Since the beginning of the pandemic, Brazil has been affected by different COVID-19 infection waves, generally related to the introduction and circulation of a new variant capable of altering the speed of transmission of the disease [49]. As seen in early 2022, the exponential increase of SARS-CoV-2 positive cases in Brazil [2] was due to the Omicron VOC introduction, first detected in São Paulo on 30 November 2022 [17,50].

Recently, our surveillance program described the introduction of Delta VOC in Brazil on 1 June 2021, in Rio de Janeiro and its rapid expansion in Brazilian territory, which reached more than 90% of the prevalence sixteen weeks later [28]. According to these previous data, Delta VOC was already predominant in all the evaluated Brazilian capitals in epidemiological week 35/2021 [28]. Our results presented here corroborate our previous results, which suggested that Delta VOC was the most representative variant until epidemiological week 50/2021 in most Brazilian capitals. The arrival of Omicron VOC caused another important change in the epidemiological scenario, culminating in the replacement of the Delta variant by Omicron in approximately 3.5 weeks (Figure 2A), faster than what was observed during Gamma-Delta [9].

Considering the emergence period of the Omicron variant and its introduction in Brazil, we identified in our data its arrival in epidemiological week 49 in Belo Horizonte, on a sample collected on 10 December 2021. In the following epidemiological week, we observed a dispersion of Omicron VOC in other capitals, such as Brasília, Goiânia, Rio de Janeiro, and São Paulo. Our results showed that the Southeast (Belo Horizonte, Rio de Janeiro e São Paulo) and Midwest (Goiânia) regions were the first affected by Omicron VOC, reaching a dominance plateau in mid-December 2021. These cities comprise the main Brazil gateways for foreigners, which may explain why the Omicron VOC arrived first in these locations [51]. The other evaluated regions (the north and northeast) reached a plateau shortly afterward, in January 2022.

The SGTF detection method used in this study allowed the identification of SGTF samples from epidemiological week 36 onwards. The SGTF samples identified before epidemiological week 49 possibly indicate the presence of the Alpha VOC, as previously described [28]. The Omicron (sublineages BA.1, BA.4, and BA.5) and Alpha VOCs share the 69–70 deletion in the Spike protein that prevents the oligonucleotide probe from binding to its target sequence, leading to what has been termed SGTF [25]. Due to the high accuracy of the SGTF method, this strategy was widely used for monitoring these variants [8,25]. Although the Omicron and Alpha VOCs share this similarity, historically, Alpha has remained at a low proportion among the circulating variants in Brazil and without overlapping dates or epidemiological weeks [9]. In contrast, the entry of the Omicron variant into the country caused an expressive increase in the number of cases, coinciding with an increase in the proportion of SGTF variants detected (Figure 2A,B). Although we observed that the Omicron VOC viral load is smaller than Delta VOC, the significant increase in the number of cases can be explained by superspreading events such as Christmas and New Year’s celebrations, in addition to the drop in the use of protective measures [52,53,54,55,56].

In early February 2022, the BA.2 sublineage, which lacks the 69–70 deletion, was inserted in Brazil [57]. In this period, we also identified a trace of this underlining that was included in the non-SGTF group. The SARS-CoV-2 variants and sublineages were detected in a subset by whole-genome sequencing. Our phylogenetic reconstruction indicated that the most representative variant of the dataset was Omicron VOC BA.1, followed by Omicron VOC BA.2 in the analyzed period. These data corroborate with the emergence of variants reported in Brazil and worldwide in the evaluated period [17,58]. Nevertheless, although the BA.2 sublineage was reported on February 8, 2022, in São Paulo [59], in our study it was detected on February 7, 2022, in Belo Horizonte. Thus, we were able to demonstrate the introduction of this sublineage before it was principally reported [59]. This finding reinforces the importance of genomic surveillance for monitoring emerging variants.

Enhanced transmissibility has been related to higher viral loads in the upper respiratory tract, as described for the previous Alpha and Delta VOCs [60,61]. The Omicron VOC is known to have higher infectivity than other variants [17,62], having quickly spread and established itself in Brazil, replacing the Delta VOC in a shorter time than other transitions previously. This phenomenon could be associated with a high viral load in patients infected with the Omicron VOC. To test this hypothesis, we evaluated the dynamics of viral loads measured from RT-qPCR Ct values.

Linear regression modeling showed a higher Ct value for patients infected by Omicron compared to patients infected by the Delta VOC, indicating a significant correlation between increased frequency of Omicron VOC and decreased viral load. Our analysis also showed a slight increase in the Ct value for the internal control (MS2), detecting the existence of random variations during the RT-qPCR diagnostic tests, although its effect is approximately 5-fold less than calculated for viral targets. However, we observed lower Ct values associated with higher viral loads in RT-qPCR tests performed in early January 2022, coinciding with superspreading events, with no variance in the MS2 exogenous Ct values in the same period. Those dates agree with the introduction of the BA.1 Omicron subvariant, which explained the third wave of COVID-19 during this period, showing the highest number of cases and transmission in Brazil [2,48]. Our findings are consistent with recent reports suggesting that the increased transmissibility of Omicron VOC is not due to a high viral load in the upper respiratory tract [63,64,65]. It is probably related to other mechanisms than higher viral excretion, such as increased affinity to the receptor of human cells and decreased sensitivity to immunity due to the many mutations of the spike protein [62,66,67].

It was also suggested that the Omicron VOC has lower virulence when compared with the Delta VOC [68]. To assess this tendency, we evaluated records from hospitalized patients from the Brazilian SRAG database in periods dominated by Delta or Omicron VOCs. The clinical data evaluated from COVID-19 patients showed that the Omicron VOC had less or equal severity to the Delta VOC. This result agrees with data from previous studies that also described a decrease in severe cases due to Omicron VOC infection [69,70,71].

## 5. Conclusions

Brazil was marked by different epidemiological scenarios with four waves of COVID-19 infection, reaching a large proportion in the number of infections and deaths. These waves were always attached to the emergence of new VOCs that allowed the maintenance and circulation of the virus in the country. In this way, the establishment of monitoring activities and the characterization of circulating variants are important for the development of public health strategies that can be employed to contain the COVID-19 pandemic.

The results of this study represented the continuity of a SARS-CoV-2 national surveillance program. Here we describe the monitoring study between August 2021 and March 2022 that detected the introduction and fast establishment of Omicron VOC in Brazil in late 2021. The continental dimensions of the country and different public health policies represent challenges in tracking the evolution and dissemination of VOCs. Nevertheless, our program was able to generate data that could provide support to public authorities, which would contribute to controlling strategies for the COVID-19 pandemic, reinforcing the importance of the creation and maintenance of nationwide surveillance programs, not only for SARS-CoV-2 but also for other infectious diseases.

## Data Availability

All generated genome sequences have been deposited on GISAID (GISAID Identifier: EPI_SET_221201my).

## Supplementary Materials

The following supporting information can be downloaded at: https://www.mdpi.com/article/10.3390/v15041017/s1, File S1: Dataset and scripts used in the genotyping analysis of SARS-CoV-2 variants; File S2: Dataset and code used in lineage classification and phylogenetic analysis; File S3: RT-qPCR Ct data and script used in this study; File S4: Symptomatology data and the script used in meta-analysis; File S5: GISAID acknowledgment table; Table S1: GISAID access number for consensus genome sequences characterized in this study; Table S2: Delta and Omicron VOC dominance periods in each capital; Table S3: Metadata, sequencing statistics, and lineage classification for all samples characterized through genome sequencing; Table S4: Lineage effect on Ct value at the national level and for each capital using a linear regression model.
