# Supplementary material for: Epidemiological Surveillance Reveals the Rise and Establishment of the Omicron SARS-CoV-2 Variant in Brazil
Source: Viruses. 2023 Apr 20;15(4):1017. doi: 10.3390/v15041017 (PMC10145299; doi:10.3390/v15041017)
Supplement: Supplementary file 1 [file viruses-15-01017-s001.zip › File_S5/File S5.pdf]

SUPPLEMENTAL TABLE

Data Availability

GISAID Identifier:	EPI_SET_221201my
doi:	10.55876/gis8.221201my

All genome sequences and associated metadata in this dataset are published in GISAID's EpiCoV database. To view the contributors of each individual sequence with details such as accession number, Virus name, Collection date, Originating Lab and Submitting Lab and the list of Authors, visit 10.55876/gis8.221201my

Data Snapshot

- EPI_SET_221201my is composed of 1,531 individual genome sequences.
- The collection dates range from 2021-11-29 to 2022-11-22;
- Data were collected in 147 countries and territories;
- All sequences in this dataset are compared relative to hCoV-19/Wuhan/WIV04/2019 (WIV04), the official reference sequence employed by GISAID (EPI_ISL_402124). Learn more at https://gisaid.org/WIV04.
