# Supplementary material for: Epidemiological Surveillance Reveals the Rise and Establishment of the Omicron SARS-CoV-2 Variant in Brazil
Source: Viruses. 2023 Apr 20;15(4):1017. doi: 10.3390/v15041017 (PMC10145299; doi:10.3390/v15041017)
Supplement: Supplementary file 1 [file viruses-15-01017-s001.zip › Table_S2.pdf]

Table S3: Periods dominated by different VOCs for each capital. The lineages showed a frequency above 90% between the initial and final dates. Intermediate frequency periods were excluded from the dataset.

**Capital**	**VOC Delta**		**VOC Omicron**	
	Date (epidemiological week)		Date (epidemiological week)	
	Start	End	Start	End
Belém	29-Ago-2021 (35)	19-Dec-2021 (51)	09-Jan-2022 (02)	27-Feb-2022 (09)
Belo Horizonte	29-Ago-2021 (35)	05-Dec-2021 (49)	26-Dec-2021 (52)	27-Feb-2022 (09)
Boa Vista	12-Sep-2021 (37)	12-Dec-2021 (50)	02-Jan-2022 (01)	06-Feb-2022 (06)
Brasília	29-Ago-2021 (35)	12-Dec-2021 (50)	02-Jan-2022 (01)	20-Feb-2022 (08)
Fortaleza	29-Ago-2021 (35)	28-Nov-2021 (48)	02-Jan-2022 (01)	27-Feb-2022 (09)
Goiânia	29-Ago-2021 (35)	28-Nov-2021 (48)	26-Dec-2021 (52)	27-Feb-2022 (09)
Macapá	10-Oct-2021 (41)	19-Dec-2021 (51)	09-Jan-2022 (02)	20-Feb-2022 (08)
Manaus	29-Ago-2021 (35)	28-Nov-2021 (48)	02-Jan-2022 (01)	13-Feb-2022 (07)
Palmas	29-Ago-2021 (35)	19-Dec-2021 (51)	09-Jan-2022 (02)	27-Feb-2022 (09)
Porto Velho	29-Ago-2021 (35)	12-Dec-2021 (50)	02-Jan-2022 (01)	27-Feb-2022 (09)
Rio de Janeiro	29-Ago-2021 (35)	21-Nov-2021 (47)	19-Dec-2021 (51)	20-Feb-2022 (08)
São Paulo	29-Ago-2021 (35)	28-Nov-2021 (48)	12-Dec-2021 (50)	27-Feb-2022 (09)
