# Supplementary material for: Epidemiological Surveillance Reveals the Rise and Establishment of the Omicron SARS-CoV-2 Variant in Brazil
Source: Viruses. 2023 Apr 20;15(4):1017. doi: 10.3390/v15041017 (PMC10145299; doi:10.3390/v15041017)
Supplement: Supplementary file 1 [file viruses-15-01017-s001.zip › Table_S4.pdf]

Table S5: Variant effect on Ct value for each capital and at the national level using a linear regression model (β). Two distinct viral targets (N and ORFab) and an internal process control (MS2) were analyzed. Positive β values indicate an increase in the Ct score in the period dominated by the Omicron VOC.

**Capital**	**Number of Samples**	**N gene**			**ORF1ab gene**			**MS2 control**		
		**β**	**95% CI**	**p-value**	**β**	**95% CI**	**p-value**	**β**	**95% CI**	**p-value**
Belém	2861	0.323	±0.757	0.403	0.468	±0.764	0.230	1.747	±1.506	6.47e-16*
Belo Horizonte	46266	1.077	±0.161	<2e-16*	1.172	±0.160	<2e-16*	0.123	±0.085	0.004*
Boa Vista	444	1.080	±2.584	0.413	1.021	±2.638	0.448	1.238	±1.332	0.069
Brasília	2976	0.448	±0.461	0.057	0.882	±0.454	0.00014*	0.621	±0.206	3.78e-09*
Fortaleza	337	-2.311	±2.985	0.130	-2.545	±3.006	0.098	-0.680	±1.258	0.290
Goiânia	3093	1.612	±0.702	6.98e-06*	1.756	±0.702	1.02e-06*	-0.480	±0.307	0.002*
Macapá	1027	2.108	±2.027	0.0418*	2.151	±2.057	0.0407*	0.008	±1.023	0.987
Manaus	565	-0.297	±2.207	0.792	-0.272	±2.246	0.813	2.129	±1.129	0.0002*
Palmas	1267	2.087	±0.767	1.15e-07*	2.148	±0.777	7.3e-08 *	1.333	±0.489	1.1e-07*
Porto Velho	2895	0.736	±0.784	0.066	0.886	±0.791	0.0281*	0.584	±0.421	0.007*
Rio de Janeiro	6383	1.382	±0.380	1.19e-12*	1.663	±0.383	<2e-16*	-0.178	±0.189	0.066
São Paulo	4771	3.318	±0.458	<2e-16*	3.645	±0.462	<2e-16*	0.495	±0.222	1.23e-05*
**Brazil**	**72885**	**1.114**	**±0.125**	**<2e-16***	**1.304**	**±0.124**	**<2e-16***	**0.267**	**±0.064**	**5.98e-16***

*Statistically significant values (α = 0.05).
